# Spray-Dried Eugenol Microparticles: Physicochemical Characterization and Enhanced Antibacterial Activity

**DOI:** 10.3390/ph19081285

**Published:** 2026-08-14

**Authors:** Vicenta Albarral Ávila, Anna Nardi-Ricart, Aitor Caballero-Román, Lara Martínez Pettina, David Miñana-Galbis, Montserrat Miñarro Carmona

**Affiliations:** 1 Microbiology Section, Department of Biology, Healthcare and Environment, Faculty of Pharmacy and Food Sciences, Universitat de Barcelona, Av Joan XXIII, 27–31, 08028 Barcelona, Catalonia, Spain; valbarral@ub.edu (V.A.Á.); lmartipe235@alumnes.ub.edu (L.M.P.); 2 Institut de Recerca de la Biodiversitat (IRBio), Universitat de Barcelona, Av Diagonal, 643, 08028 Barcelona, Catalonia, Spain; 3 Pharmacy, Pharmaceutical Technology, and Physico-Chemical Department, Universitat de Barcelona, Av. Joan XXIII, 27–31, 08028 Barcelona, Catalonia, Spain; caballeroaitor@ub.edu (A.C.-R.); minarromontse@ub.edu (M.M.C.); 4 IDIBELL-UB Research Group, Pharmacotherapy, Pharmacogenomics and Pharmaceutical Technology, Avinguda Granvia, 199–203, L’Hospitalet de Llobregat, 08908 Barcelona, Catalonia, Spain

**Keywords:** eugenol, microencapsulation, spray drying, antimicrobial resistance, minimum inhibitory concentration, biopolymers

## Abstract

**Background/Objectives**: Antimicrobial resistance is a critical threat to global public health. Eugenol is a bioactive compound with broad-spectrum antimicrobial activity that has attracted increasing interest as a naturally derived antimicrobial agent with potential complementary applications to conventional antibiotics, but its clinical application is severely limited by its high volatility, low water solubility and thermo-oxidative instability. The main objective of this study was to develop eugenol-loaded microparticles using a ternary biopolymer matrix, to characterise their main physicochemical properties, and to evaluate their in vitro antimicrobial efficacy against clinically relevant bacterial reference strains. **Methods:** The microparticles were formulated from an emulsion of maltodextrin, gum arabic and soy lecithin, and encapsulated using a spray-drying technique. Product recovery, particle morphology assessed by scanning electron microscopy (SEM), particle size distribution determined by laser diffraction, and encapsulation efficiency quantified by GC-FID were analysed. Subsequently, antimicrobial activity was evaluated by comparing the microparticles with free eugenol using agar well diffusion and broth microdilution assays to determine the minimum inhibitory concentration (MIC) against eight bacterial strains. **Results:** The spray-drying process achieved a product recovery of 61.88% and an encapsulation efficiency of 52.45%. The resulting microparticles exhibited a smooth, spherical morphology with diameters of less than 20 µm. In microbiological assays, microencapsulation significantly reduced MIC values by 4- to 16-fold compared with free eugenol for susceptible strains. The formulation exhibited potent activity against most of the Gram-positive and Gram-negative pathogens tested, except for *Pseudomonas aeruginosa*, which remained resistant to both formulations. **Conclusions:** The encapsulation of eugenol in this optimised biopolymer matrix substantially improved its antimicrobial efficacy against the tested bacterial strains. These findings highlight the potential of spray-dried eugenol microparticles as a promising antimicrobial formulation and provide a basis for their further development for topical applications. Further studies are warranted to evaluate their pharmaceutical performance and antimicrobial mechanisms.

## 1. Introduction

The growing incidence of antimicrobial resistance (AMR) constitutes one of the most critical threats to global public health and poses a systemic challenge to modern medicine. Epidemiological projections warn that, unless effective interventions are implemented, AMR could cause ten million deaths annually by 2050 [1]. The rapid spread of multidrug-resistant (MDR) pathogens, classified by the World Health Organization (WHO) as “priority pathogens”, including methicillin-resistant *Staphylococcus aureus* (MRSA) and carbapenem-resistant *Enterobacterales*, has created an urgent need to develop alternative and truly sustainable antimicrobial strategies [2,3].

At the same time, the clinical development pipeline for conventional antibiotics is experiencing severe stagnation, evidenced by the scarcity of new drug classes approved in recent decades [4]. This critical gap has redirected pharmaceutical research towards bioactive phytochemicals, many of which exhibit multi-target mechanisms of action that may reduce the likelihood of resistance development [5]. In this context, eugenol (4-allyl-2-methoxyphenol), the major phenylpropanoid constituent of clove essential oil (*Syzygium aromaticum*), has emerged as a leading bioactive candidate with demonstrated broad-spectrum antimicrobial activity [6].

The antimicrobial activity of eugenol lies in its structural configuration: the lipophilic benzene ring facilitates its interaction with the bacterial lipid bilayer, whilst the phenolic hydroxyl group disrupts cellular integrity [7]. Unlike single-target antibiotics, eugenol exerts pleiotropic effects, such as membrane depolarisation, ion channel blockade, intracellular ATP depletion and the induction of reactive oxygen species (ROS), which have been associated with bacterial DNA damage [8,9]. Furthermore, at sub-inhibitory concentrations, eugenol has also been reported to interfere with virulence-related traits, including quorum sensing systems and biofilm formation [10].

Despite its undeniable therapeutic potential, the clinical translation of this important bioactive compound is severely limited by its adverse physicochemical properties: extreme volatility, poor aqueous solubility (less than 0.1% *w*/*v*), intense aroma and marked susceptibility to thermo-oxidative and photolytic degradation [11,12].

To overcome these technological barriers, pharmaceutical engineering strategies—specifically microencapsulation via spray drying—represent a proven and robust technological solution [13]. This process facilitates the entrapment of eugenol within biopolymer matrices, significantly improving its thermodynamic stability, masking its intrinsic odour and facilitating its incorporation into pharmaceutical formulations [14,15].

Recent advances in encapsulation technology have demonstrated the effectiveness of natural polymer blends for eugenol delivery [16]. The synergistic combination of maltodextrin (MD) as a structuring agent, gum arabic (GA) as an emulsifier and soy lecithin (SL) as an interfacial stabiliser yields solid microparticles with high encapsulation efficiency and controlled release properties for hydrophobic bioactive compounds [17,18].

Current reports on encapsulated essential oils often demonstrate limited efficacy against Gram-negative pathogens due to the impermeability of their lipopolysaccharide (LPS) outer membrane [18]. This study distinguishes itself by strategically incorporating soy lecithin into a ternary biopolymer matrix (maltodextrin:gum arabic:soy lecithin). Unlike previous formulations where lecithin is merely utilized as a technological excipient, it is postulated that its amphiphilic nature provides a specific mechanistic advantage: it acts as an interfacial permeabilizer that enhances the interaction between the solid eugenol-loaded microparticles and the bacterial lipid bilayer. This formulation advances the current state of knowledge by bridging the gap between phytochemical efficacy and pharmaceutical applicability through three main objectives: first, to develop optimised eugenol-loaded microparticles using a ternary biopolymer matrix; second, to comprehensively characterise their main physicochemical properties; and third, to systematically evaluate their antimicrobial efficacy against clinically relevant bacterial reference strains through comparative in vitro assays.

## 2. Results

### 2.1. Eugenol Microparticles Analysis

#### 2.1.1. Product Recovery

The product recovery of the eugenol microparticles after the spray-drying process was 61.88%.

#### 2.1.2. Particle Size Distribution

**Figure 1:**
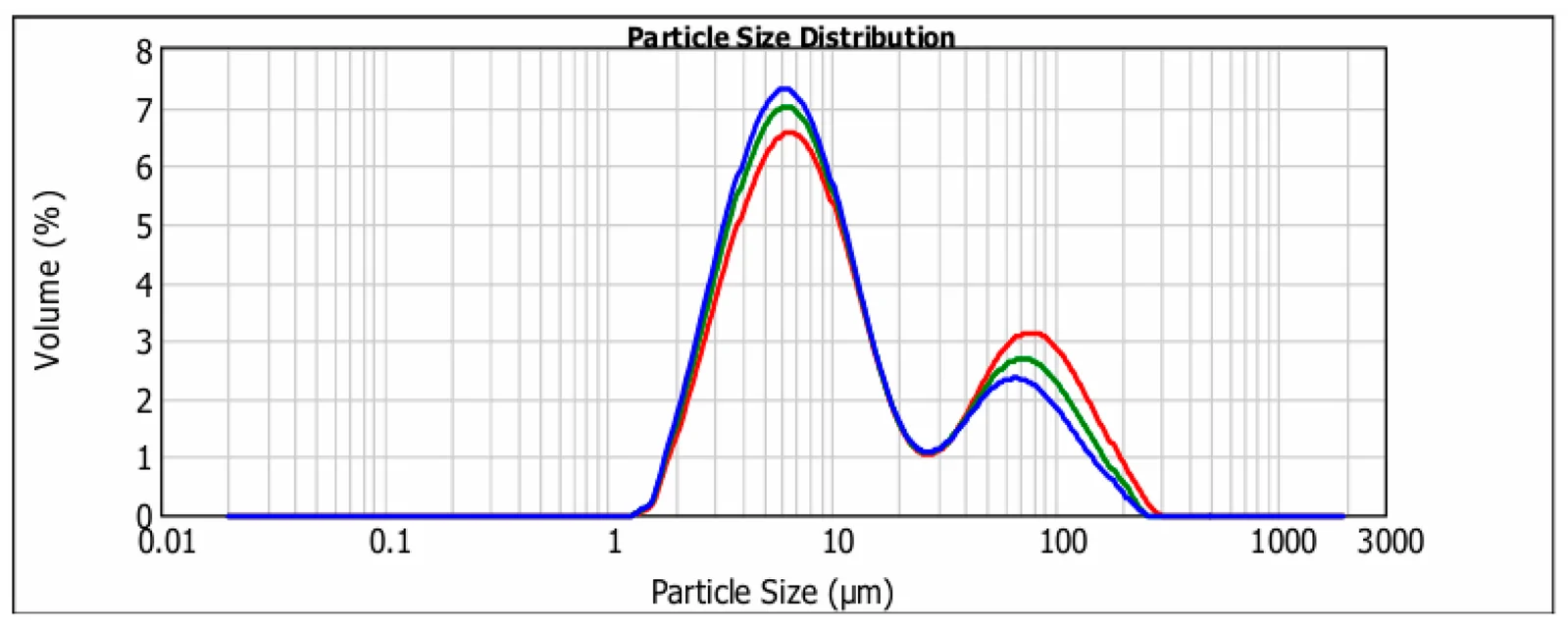
Particle size Distribution of Eugenol microparticles. Each color represents the result of one of a total of three independent measurements.

The volume-based particle size distribution was characterized by D_10_, D_50_ and D_90_ values of 3.078 µm, 7.871 µm, and 71.490 µm, respectively. Figure 1 shows the graphic representation of Particle Size Distribution.

The corresponding Span, calculated as (D_90_ − D_10_)/D_50_, was 8.69, indicating a broad particle size distribution. The d[3,2] and d[4,3] values were 6.589 and 22.760 µm, respectively. Values represent the mean ± SD of three independent measurements.

This relatively high Span value is consistent with the bimodal distribution profile observed, displaying a primary population of fine particles and a secondary population of larger agglomerates (Figure 1).

#### 2.1.3. Particle Morphology

**Figure 2:**
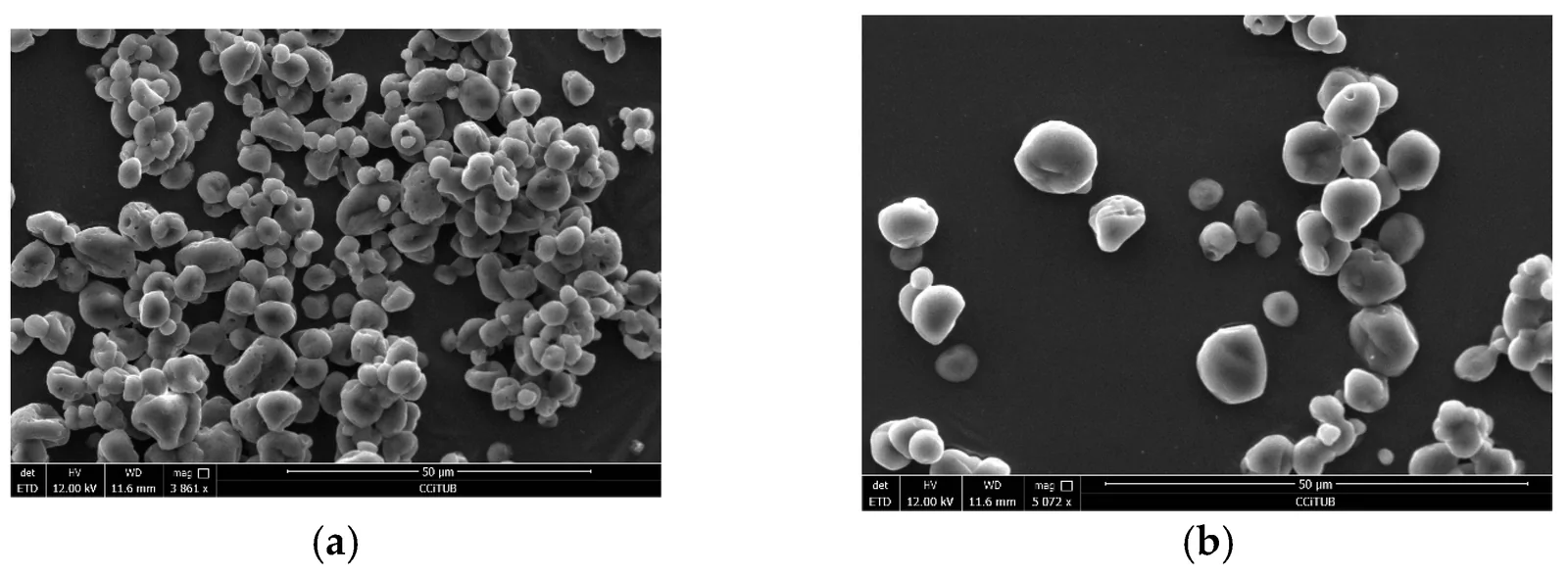
Eugenol microparticles viewed under SEM, showing a general overview; (**a**) Observed with 6074× magnification; (**b**) Observed with a 5072× magnification.

Eugenol microparticles had a smooth spherical shape with a diameter of less than 20 µm as can be observed in Figure 2.

#### 2.1.4. Eugenol Encapsulation Efficiency

To determine the encapsulated EUG content, the microparticles were initially dissolved in Milli-Q^®^ water, followed by a hexane extraction step, with the final quantification performed via GC-FID.

The Encapsulation efficiency was 52.45%.

#### 2.1.5. Moisture Content

The moisture content was 7.05%.

#### 2.1.6. Hygroscopiciy

The hygroscopicity value was 13.9%.

### 2.2. Eugenol Microparticles Microbiological Analysis

#### 2.2.1. Agar Well Diffusion Assay

The antimicrobial activity of free eugenol (EUG) and microencapsulated eugenol (EUG-MC) was evaluated against eight bacterial reference strains: *Bacillus spizizenii* ATCC 6633, *Enterococcus faecalis* ATCC 29212, *Escherichia coli* ATCC 25922, *Klebsiella pneumoniae* ATCC 13883^T^, *Listeria monocytogenes* ATCC 15313^T^, *Pseudomonas aeruginosa* ATCC 27853, *Salmonella enterica* ATCC 14028 and *Staphylococcus aureus* ATCC 25923.

Both formulations (EUG and EUG-MC) exhibited concentration-dependent antimicrobial activity against the tested strains (Table 1). Statistical analysis confirmed a significant effect of both the formulation type and the concentration, as well as a significant interaction between these two factors (*p* < 0.05). Free eugenol consistently produced larger zones of inhibition across all concentrations. For instance, against *S. aureus* at 250 mg/mL, free eugenol produced an inhibition zone of 18.7 ± 0.6 mm, while EUG-MC produced 15.9 ± 0.3 mm, a difference that was statistically significant. This pattern was observed for all susceptible strains and reflects the faster diffusion kinetics of solubilized eugenol compared to particulate microparticles that require hydration and dissolution in the agar matrix [19].

Gram-positive bacteria demonstrated greater susceptibility, with *S. aureus* being the most sensitive, followed by *L. monocytogenes* and *B. spizizenii* [20]. Gram-negative bacteria showed variable sensitivity, with *E. coli*, *S. enterica*, and *K. pneumoniae* being susceptible, while *P. aeruginosa* exhibited complete resistance to free eugenol and only marginal sensitivity to EUG-MC at the highest concentration (6.9 ± 1.0 mm).

Control assays validated the experimental setup. No growth was observed in sterility controls, while growth controls showed confluent lawns. The positive controls, tetracycline (TE30) and levofloxacin (LV5), produced inhibition zones consistent with standard susceptibility profiles for each strain.

#### 2.2.2. Minimum Inhibitory Concentration (MIC)

The broth microdilution assay revealed a significant enhancement of antimicrobial activity through microencapsulation (Table 2). The microencapsulated formulation (EUG-MC) demonstrated MIC values 4- to 16-fold lower than those of free eugenol against susceptible strains [18]. The reduction in MIC achieved by microencapsulation was statistically significant for each individual susceptible strain (*p* < 0.05).

EUG-MC exhibited MIC values ranging from 0.5 to 2 mg/mL (0.05–0.2%), while free eugenol required concentrations between 4 and 16 mg/mL (0.4–1.6%) for inhibition. The most substantial improvement was observed against *B. spizizenii*, with a 16-fold reduction in MIC (from 16 mg/mL for free oil to 1 mg/mL for microparticles). Against *L. monocytogenes*, an 8-fold reduction was achieved (from 4 mg/mL to 0.5 mg/mL).

The formulation showed potent activity against both Gram-positive (*S. aureus*, *E. faecalis*, *B. spizizenii*, *L. monocytogenes*) and Gram-negative (*E. coli*, *S. enterica*, *K. pneumoniae*) bacterial strains. Consistent with diffusion assay results, *P. aeruginosa* remained resistant to both formulations, with no inhibitory activity observed at the highest tested concentration (62.5 mg/mL).

All control wells performed as expected. The reference antibiotics tetracycline and levofloxacin yielded MIC values within the documented ranges for the ATCC strains used, confirming the validity of the assay conditions (Table 3).

## 3. Discussion

This study demonstrates that microencapsulation of eugenol using a ternary biopolymer matrix significantly enhances its antimicrobial efficacy against clinically relevant bacterial pathogens. The 4- to 16-fold reduction in MIC values represents a substantial improvement over free eugenol and is consistent with previous reports on phytochemical delivery systems [16].

### 3.1. Enhanced Efficacy Through Optimized Encapsulation

The superior performance of EUG-MC in the broth microdilution assay, despite producing smaller inhibition zones in agar diffusion, exemplifies the methodological discrepancy often observed with particulate delivery systems [19]. This disparity can be attributed to fundamental differences in the test environments. In the solid agar matrix, free eugenol diffuses readily, whereas the particulate microparticles experience a kinetic lag due to the requisite steps of hydration and dissolution before the active compound is released. Conversely, in liquid broth, the ternary biopolymer matrix provides several advantages for antimicrobial delivery. Maltodextrin serves as an effective carrier with optimal drying properties, gum arabic provides stabilization of the initial oil-in-water emulsion, and lecithin improves interfacial activity, potentially facilitating closer contact between eugenol-loaded microparticles and bacterial cells [17,18].

The formulation showed broad-spectrum activity against both Gram-positive and Gram-negative pathogens is a key finding. For Gram-positive bacteria, which lack a protective outer membrane, the liberated eugenol can directly interact with the phospholipid bilayer, inducing membrane disruption and the subsequent leakage of intracellular components [21]. This mechanism aligns with the pronounced susceptibility observed for *Staphylococcus aureus* and *Listeria monocytogenes*.

Compared to recently published eugenol delivery systems, which often rely on simple binary polymer blends and subsequently require significantly higher concentrations to inhibit Gram-negative species [18], this ternary matrix design specifically overcomes this limitation. The potent activity demonstrated against Gram-negative species *Escherichia coli*, *Salmonella enterica*, and *Klebsiella pneumoniae* is particularly noteworthy, as their outer membrane, rich in lipopolysaccharide (LPS), typically constitutes a formidable permeability barrier to hydrophobic molecules [22]. The efficacy of the formulation against these pathogens suggests a critical role for the lecithin component. Its amphiphilic nature likely facilitates initial interaction with and destabilization of the outer membrane, enhancing eugenol penetration in a manner consistent with observations in other emulsified antimicrobial systems [18].

From a technological perspective, the achieved encapsulation efficiency (EE) of 52.45% is considered moderate for general solid pharmaceuticals, but is highly acceptable and consistent with previous reports for the spray-drying of volatile essential oils. The specific loss of eugenol during processing could be attributed to its intrinsic high volatility and sensitivity to thermo-oxidative conditions. During atomization at an inlet temperature of 130 °C, the rapid evaporation of water from the droplet surface facilitates a concurrent co-evaporation of the dispersed eugenol. Furthermore, the active compound that partitions to the outer surface of the polymer matrix before crust formation is inevitably lost to the exhaust air stream.

Alongside the encapsulation efficiency, the physical characteristics of the resulting powder further reflect the complexity of the atomization process. The particle size distribution was broad, as demonstrated by the marked difference between D_10_, D_50_, and D_90_, and by the calculated span of 8.69. The coarse fraction detected by laser diffraction may represent larger particles, agglomerates, or both. This interpretation is consistent with the discrepancy between the predominantly sub-20 µm individual particles observed by scanning electron microscopy (SEM) and the substantially larger D_90_ obtained by laser diffraction. SEM and laser diffraction provide different and complementary information [23]: SEM visualizes a limited number of dried particles within selected microscopic fields, whereas laser diffraction provides a volume-weighted equivalent-sphere distribution of the dispersed powder. Consequently, a relatively small number of large particles or agglomerates may markedly increase the D_90_ and the upper tail of the volume-based distribution. While laser diffraction is an appropriate technique for the size range observed here, its interpretation requires careful consideration of the optical model and dispersion conditions.

This broad distribution may also reflect variability in droplet formation during atomization or incomplete optimization of the feed and spray-drying parameters. However, the present measurements do not allow these potential causes to be definitively distinguished from agglomeration occurring after drying or during sample preparation.

While the current parameters successfully yielded a functional and highly active antimicrobial powder, further process optimization is warranted. Future studies will systematically optimize this formulation by implementing a rigorous Quality by Design (QbD) framework. This approach will be utilized to define the optimal design space, specifically investigating the critical interactions between higher total solid contents, altered core-to-wall ratios, and lower inlet temperatures to maximize eugenol retention without compromising the moisture content and flowability of the final microparticles.

### 3.2. Pseudomonas aeruginosa Resistance: Understanding the Limitations

*P. aeruginosa* resistance to both formulations is consistent with its well-documented intrinsic resistance mechanisms [24,25]. These mechanisms primarily involve a multi-factorial defense strategy. First, the pathogen possesses a low-permeability outer membrane with specific porin restrictions that limit the entry of antimicrobials. Second, it constitutively expresses potent efflux pumps, most notably the MexAB-OprM system, which actively extrudes hydrophobic compounds like eugenol. Third, *P. aeruginosa* exhibits adaptive stress responses capable of modifying its membrane lipid composition, further reducing susceptibility [26,27].

These findings align with studies showing limited efficacy of essential oils against *P. aeruginosa* without adjuvant strategies [28]. Future formulations may benefit from incorporating efflux pump inhibitors (e.g., phenylalanine-arginine β-naphthylamide) or membrane permeabilizers (e.g., EDTA) to overcome these barriers [29].

### 3.3. Pharmaceutical Implications and Future Directions

The achievement of MIC values as low as 0.5 mg/mL (0.05%) against key bacterial pathogens within the tested range supports the further pharmaceutical development of this formulation and its future evaluation for topical antimicrobial applications, in line with recent evidence showing that eugenol-enriched polymeric scaffolds can combine antibacterial and anti-biofilm activity with wound-healing potential [30].

The promising antimicrobial activity demonstrated by the spray-dried eugenol microcapsules supports their further pharmaceutical development and their future incorporation into topical dosage forms.

To achieve optimal translational and pharmaceutical performance, the incorporation of these microparticles into specific topical dosage forms must consider both the physicochemical nature of the ternary biopolymer matrix and its particle size distribution.

Given their micrometric dimensions, the particles are not expected to cross intact skin as complete structures; rather, their intended function is to remain within the topical vehicle or on the skin surface. Following incorporation into an appropriate pharmaceutical dosage form, they may potentially act as reservoirs from which eugenol could be gradually released upon hydration of the polymeric matrix.

The redispersion and release behaviour may also be influenced by the particle size distribution. Smaller particles, owing to their higher surface-area-to-volume ratio, may hydrate more rapidly than larger particles, potentially contributing to differences in the release profile of eugenol. However, these hypotheses require experimental confirmation through dedicated release studies.

Furthermore, the release profile is expected to depend on the selected formulation vehicle. Incorporation into aqueous-based systems, such as hydrogels or oil-in-water (O/W) creams, may promote hydration of the hydrophilic wall materials (maltodextrin and gum arabic), potentially facilitating the gradual release of eugenol. Alternatively, incorporation into anhydrous semi-solid bases, polymeric films, or dry wound dressings may help preserve the structural integrity of the microparticles during storage until exposure to physiological moisture. These formulation-dependent behaviours could represent potential advantages for topical antimicrobial therapy; however, they remain hypothetical and should be confirmed through drug release, stability, ex vivo skin permeation, and in vivo efficacy studies using the final pharmaceutical dosage forms.

Compared with conventional antibiotics, eugenol’s multi-target mechanism may reduce the likelihood of resistance development; however, combination therapies, particularly with colistin, should be further explored to assess potential synergistic effects [31]. Future studies should also investigate in vivo efficacy, pharmacokinetic profiles, and the feasibility of large-scale manufacturing.

Additionally, comprehensive physicochemical characterization using Fourier-transform infrared spectroscopy (FTIR), X-ray diffraction (XRD), and differential scanning calorimetry (DSC) will be performed in future preformulation studies to further elucidate the interactions within the ternary matrix and the solid-state properties of encapsulated eugenol. Likewise, long-term storage stability studies conducted in accordance with ICH Q1 guidelines will be essential to determine the shelf-life, physical integrity, and retention of the encapsulated volatile compound prior to its incorporation into final topical dosage forms.

## 4. Materials and Methods

### 4.1. Materials and Bacterial Strains

Eugenol (EUG, 99% purity) was kindly provided by Lidervet (Tarragona, Catalonia, Spain). Maltodextrin (MD, C-Dry™ MD, dextrose equivalent 12–16) and soy lecithin (SL, Emultop™ IP) were supplied by Cargill (Barcelona, Catalonia, Spain). Spray-dried gum arabic (GA) derived from acacia trees was obtained from Merck (Darmstadt, Germany).

Distilled water was utilized to prepare the polymer solutions, whereas Milli-Q^®^ purified water (Merck Millipore Advantage A10 system, Burlington, MA, USA) was specifically employed to dissolve the microparticles during analytical assays. Hexane (analytical grade alkane mixture) and pharmaceutical-grade 2-propanol were sourced from Panreac AppliChem (ITW Reagents, Barcelona, Catalonia, Spain); 99% pure azulene was purchased from Alfa Aesar (Haverhill, MA, USA).

Mueller–Hinton broth (MHB) and Mueller–Hinton agar (MHA), used for antimicrobial susceptibility testing, were obtained from Oxoid Ltd. (Basingstoke, UK). Bacterial strains were recovered from cryobeads or frozen stocks stored at −80 °C in Tryptic Soy Broth (TSB) (Oxoid Ltd.) supplemented with 20% (*v*/*v*) glycerol (Panreac, Barcelona, Catalonia, Spain). For strain recovery, cryobeads or frozen aliquots were streaked onto Tryptic Soy Agar (TSA) (Condalab, Madrid, Spain) and incubated at 37 °C for 24–48 h. Isolated colonies were subsequently subcultured on TSA and incubated at 37 °C for 24 h to ensure purity and obtain well-isolated colonies before antimicrobial testing. Quarter-strength Ringer’s solution was prepared from Ringer’s tablets (Oxoid Ltd.) and used to prepare bacterial suspensions for the agar diffusion assays. For MIC determinations, bacterial inocula were prepared in MHB from isolated colonies grown on TSA. Dimethyl sulfoxide (DMSO) (Sigma-Aldrich, Darmstadt, Germany) was used as the solvent for free eugenol, whereas Tween 80 (Scharlab, Barcelona, Catalonia, Spain) was used as an emulsifying agent for the preparation of the free eugenol emulsion. Resazurin sodium salt (Panreac, Barcelona, Catalonia, Spain) was prepared in sterile distilled water at a concentration of 0.1 mg/mL, sterilized by filtration through a 0.22 µm membrane filter, and used as a metabolic viability indicator for MIC determination due to the inherent turbidity of the microencapsulated eugenol suspension. Sterile flat-bottom 96-well microtiter plates were obtained from SPL Life Sciences (Pocheon, Republic of Korea). Commercial antibiotic discs containing tetracycline (30 µg) and levofloxacin (5 µg) were used as positive controls in the agar diffusion assays.

Antimicrobial activity was evaluated against eight reference strains from the American Type Culture Collection (ATCC; Manassas, VA, USA): *Bacillus spizizenii* ATCC 6633 (formerly *B. subtilis*), *Enterococcus faecalis* ATCC 29212, *Escherichia coli* ATCC 25922, *Klebsiella pneumoniae* ATCC 13883^T^, *Listeria monocytogenes* ATCC 15313^T^,, *Pseudomonas aeruginosa* ATCC 27853, *Salmonella enterica* serovar Typhimurium ATCC 14028, and *Staphylococcus aureus* ATCC 25923.

### 4.2. Eugenol Microparticles Preparation

Initially, the MD and GA polymers were allowed to hydrate in distilled water for 24 h at room temperature (24 °C). Following this period, SL was incorporated at a concentration of 0.5% (*w*/*w*) into the polymer blend, which had a 3:1 (MD:GA) ratio. To prepare the emulsion, a high-shear homogenizer (Ultra-Turrax^®^ T-25; IKA^®^-Werke, Staufen, Germany) was operated at 15,000 rpm for 5 min. Simultaneously during homogenization, the necessary volume of EUG was added dropwise to achieve a 1:1 ratio.

The atomization process was performed using a Büchi B-290 Mini Spray Drier (Büchi Ibérica; Barcelona, Catalonia, Spain) equipped with a 1.4 mm diameter nozzle. To always guarantee a homogeneous mixture, the solution was maintained under constant magnetic stirring. The operating conditions of the spray dryer were set with an inlet temperature of 130 °C, an outlet temperature of 90 °C, an atomization pressure of 4 bar, and an airflow of 80%. The feed rate was maintained at 1.5 mL/min, and the nozzle cleaner system at level 4. The total solid content of the initial emulsion was 33.33% (*w*/*w*). Finally, the resulting samples were preserved at 24 °C in airtight containers until subsequent analysis.

### 4.3. Eugenol Microparticles Analysis

#### 4.3.1. Product Recovery

To determine the efficiency of the process, the product recovery (spray-drying yield) was calculated according to Equation (1). This mathematical relationship establishes the ratio between the final dry mass of the manufactured EUG microparticles and the combined initial mass of both the core compound and the wall materials.

(1) $$\mathrm{Product}\;\mathrm{recovery}\;(\%)=\frac{\mathrm{Final}\;\mathrm{product}}{\mathrm{MD}+\mathrm{GA}+\mathrm{SL}+\mathrm{EUG}}\times 100$$

In this formula, “Final product” corresponds to the total weight of the microparticles successfully collected post-atomization. The terms MD, GA, SL, and EUG denote the starting amounts—expressed in grams—of maltodextrin, gum arabic, soy lecithin, and eugenol added to the initial formulation, respectively.

#### 4.3.2. Particle Size Distribution

Particle size distribution (PSD) analysis of microparticles was carried out utilizing the laser diffraction technique. Measurements were executed on a Mastersizer Hydro 2000 SM apparatus (Malvern Instruments; Malvern, UK) coupled with a Malvern Dispersion Unit Controller (Malvern Instruments; Malvern, UK). For the assay, approximately 1 g of the powder was suspended in 2-propanol (using a refractive index of 1.390) under constant agitation. To guarantee the reliability of the results, all tests were performed in triplicate, and data was processed via the Mastersizer 2000 software (version 5.60). The characterization of the particle size was primarily based on the 90% cumulative volume diameter, denoted as d_(0.9)_.

To ensure a well-rounded understanding of the distribution profile, the following physical parameters were also measured:

- Surface-weighted mean diameter (d[3,2]) Equation (2).

(2) $$d[3,2]=\frac{\sum n_{i}\;d_{i}^{3}}{\sum n_{i}\;d_{i}^{2}}$$

- Volume-weighted mean diameter (d[4,3]) Equation (3).

(3) $$d[4,3]=\frac{\sum n_{i}\;d_{i}^{4}}{\sum n_{i}\;d_{i}^{3}}$$

In these formulas, n_i_ stands for the frequency of particles within a given size fraction, and d_i_ represents the associated particle diameter (µm).

#### 4.3.3. Particle Morphology

To observe the surface morphology, an Environmental Scanning Electron Microscope (ESEM, Quanta 200 FEI, XTE325/D8395; FEI, Hillsboro, OR, USA) was used.

#### 4.3.4. Eugenol Encapsulation Efficiency

The encapsulation efficiency (EE%) was calculated by comparing the experimentally quantified eugenol (EUG) concentration against the theoretical payload expected within the microparticles, according to Equation (4):

(4) $$\mathrm{EE}\%\;=\frac{\mathrm{Experimental}\;\mathrm{EUG}\;\mathrm{content}}{\mathrm{Theoretical}\;\mathrm{EUG}\;\mathrm{content}}\times 100$$

Based on the initial 1:1 ratio of EUG to polymeric wall materials in the formulation, the theoretical EUG value was established at 50.00%.

To quantify the EUG content, Gas Chromatography coupled with a Flame Ionization Detector (GC-FID) was utilized, featuring azulene as the internal standard. The extraction protocol involved dispersing 0.2 g of the sample in Milli-Q^®^ water, followed by vortex mixing for 1 min and ultrasonication for 10 min. Subsequently, hexane was introduced to extract the analyte. The mixture was vortexed again and centrifuged for 3 min at 2727× *g* and 20 °C (Eppendorf 5702 R, Hamburg, Germany). The resulting supernatant was transferred into an amber volumetric flask. To guarantee complete recovery, this hexane extraction cycle was performed in triplicate. For the final analytical sample, 10 mL of the pooled extract was combined with 5 mL of the internal standard solution (3 mg/mL azulene in hexane) and diluted to volume with additional hexane before being stored in amber vials. To evaluate only the surface-adsorbed eugenol, this exact procedure was replicated without the initial aqueous dissolution step.

Chromatographic analysis was executed on a Shimadzu GC-2010 Plus instrument (Shimadzu Corporation, Kyoto, Japan) equipped with an Equity™-5 fused silica capillary column (30 m × 0.25 mm, 0.25 µm film thickness). The stationary phase consisted of 5% diphenyl and 95% dimethyl polysiloxane (Supelco^®^, Bellefonte, PA, USA). The operating parameters were set as follows: helium as the carrier gas, constant flow rate of 1.4 mL/min, an injection port temperature of 250 °C, and a detector temperature of 300 °C. The oven temperature program was designed to ramp from 100 °C to 295 °C.

#### 4.3.5. Moisture Content

The moisture content was determined using a Sartorius Moisture Analyser MA35 (Sartorius, Göttingen, Germany), based on the thermogravimetric method. Measurements were carried out at 105 °C for 5 min, using 1 g of sample spread in a uniform layer on a disposable dish.

Once the drying cycle had begun, infrared radiation evaporated the moisture from the sample until the preset time was reached, automatically recording the percentage of mass loss relative to the initial mass.

#### 4.3.6. Hygroscopicity

Hygroscopicity was determined by the percentage increase in sample weight after being kept in a humidifier at a relative humidity of 76% (±2%) and a temperature of 22 °C ± 2 °C for 24 h.

### 4.4. Eugenol Microparticles Microbiological Analysis

#### 4.4.1. Preparation of Test Samples

Stock preparations of free eugenol (EUG) and microencapsulated eugenol (EUG-MC) were prepared fresh for each assay, using distinct vehicles and concentrations according to the test format.

For the agar well diffusion assay, a concentrated stock of free EUG was first prepared by solubilizing pure eugenol in 100% dimethyl sulfoxide (DMSO), which was then diluted as needed with sterile distilled water for the assay. In parallel, EUG-MC powder was directly suspended and homogenized in sterile distilled water containing 2.5% (*v*/*v*) DMSO to facilitate dispersion and maintain comparable solvent conditions of the free eugenol sample.

For the broth microdilution assay (MIC determination), the free EUG was emulsified in Mueller–Hinton Broth (MHB) supplemented with 2% (*v*/*v*) DMSO and 2% (*v*/*v*) Tween 80 to obtain a final eugenol concentration of 125 mg/mL. In parallel, the EUG-MC powder was suspended and homogenized directly in sterile MHB to the same final equivalent eugenol concentration.

All subsequent two-fold serial dilutions for each assay were prepared from the corresponding master stocks using their respective base vehicles: sterile distilled water containing 2.5% (*v*/*v*) DMSO for agar assay dilutions, and MHB (for EUG-MC) or MHB supplemented with 2% DMSO and 2% Tween 80 (for free EUG) for broth assay dilutions.

#### 4.4.2. Inoculum Standardization

For the agar well diffusion assay, bacterial suspensions were prepared in sterile quarter-strength Ringer’s solution and adjusted to a 0.5 McFarland standard (≈1.5 × 10^8^ CFU/mL). This suspension was then diluted 1:100 in Ringer’s solution to achieve approximately 1.5 × 10^6^ CFU/mL for plate inoculation.

For the broth microdilution assay, suspensions were prepared and adjusted to the 0.5 McFarland standard directly in MHB, according to CLSI guidelines [32]. A 1:100 dilution in fresh MHB was performed, followed by a final adjustment to obtain a working inoculum of 5 × 10^5^ CFU/mL in each well of the microdilution plate.

#### 4.4.3. Agar Well Diffusion Assay

A preliminary antimicrobial screening was performed using the agar well diffusion method [33]. Mueller–Hinton agar (MHA) plates were uniformly inoculated with the standardized bacterial suspensions, containing approximately 1.5 × 10^6^ CFU/mL. Wells of 6 mm diameter were prepared in the agar and filled with 50 µL of each test sample, either free EUG or EUG-MC. Six two-fold serial dilutions prepared from the 250 mg/mL stock preparations were tested. Each condition was tested in triplicate.

Commercial antibiotic discs were used as positive controls, whereas the corresponding vehicles were included as negative controls: the DMSO-containing vehicle used for free EUG and sterile distilled water containing 2.5% (*v*/*v*) DMSO for EUG-MC. After incubation at 37 °C for 18–24 h, inhibition zone diameters were measured using a digital caliper (HBM Machines, Moordrecht, The Netherlands). All assays were performed in triplicate.

#### 4.4.4. Minimum Inhibitory Concentration (MIC) Determination

MIC values were determined by broth microdilution in sterile 96-well microtiter plates according to the CLSI M07 method [32]. Two-fold serial dilutions of each antimicrobial preparation were prepared directly in the plates from the corresponding 125 mg/mL stock preparations. Briefly, 100 µL of the stock preparation was added to the first well of each row and mixed with 100 µL of MHB. Serial two-fold dilutions were then performed by transferring 100 µL from one well to the next, each containing 100 µL of MHB. This procedure was repeated across the plate, and the excess volume was discarded from the final well to obtain a uniform volume of 100 µL per well before inoculation.

Subsequently, 100 µL of the standardized bacterial inoculum was added to each well, resulting in a final volume of 200 µL per well and a final bacterial concentration of approximately 5 × 10^5^ CFU/mL. The final tested concentration range started at 62.5 mg/mL in the first well and decreased by two-fold serial dilutions across the plate.

Plates were incubated at 37 °C for 24 h. The MIC was defined as the lowest concentration showing no visible growth, with confirmation by resazurin reduction assay (0.01% *w*/*v*) [34]. Sterility (MHB only) and growth (MHB with inoculum) controls were included in each run. All determinations were performed in triplicate.

All experiments were performed in triplicate (*n* = 3). Data are presented as mean ± standard deviation (SD). For the agar well diffusion assay, inhibition zone diameters were analyzed to evaluate the effects of formulation, concentration, and their interaction using two-way analysis of variance (ANOVA). When significant differences were detected, post hoc multiple comparisons were performed. MIC values were determined from three independent assays and expressed as mg/mL of eugenol equivalents to allow direct comparison between free EUG and EUG-MC. Differences between formulations were assessed based on changes in two-fold dilution steps.

## 5. Conclusions

Eugenol-loaded microcapsules (EUG-MC) have been successfully developed and characterised using an optimised ternary biopolymer matrix. The encapsulation process significantly enhanced the antimicrobial efficacy of eugenol, resulting in a 4- to 16-fold reduction in MIC values against priority bacterial pathogens. Whilst the formulation demonstrated potent broad-spectrum activity against both Gram-positive and Gram-negative bacteria, the resistance of *P. aeruginosa* highlights the need for adjuvant strategies. These findings support the further development of eugenol microcapsules as a promising alternative for treating multidrug-resistant infections, particularly in topical applications where conventional antibiotics face increasing limitations.

## Figures and Tables

**Table 1:** (**A**). Zone of inhibition (mm, mean ± SD) for free eugenol (oil). Concentrations expressed as mg eugenol/mL. (**B**). Zone of inhibition (mm, mean ± SD) for spray-dried microparticles. Concentrations expressed as mg eugenol/mL.

(**A**)						
**Bacterial Strain**	**250**	**125**	**62.5**	**31.2**	**16**	**8**
***B. spizizenii*** **ATCC 6633**	14.7 ± 0.9	12.4 ± 0.3	10.9 ± 0.6	9.3 ± 1.0	7.9 ± 1.0	4.5 ± 3.9
***E. coli*** **ATCC 25922**	16.6 ± 0.5	14.1 ± 0.8	11.0 ± 0.5	9.8 ± 0.4	8.3 ± 1.4	2.7 ± 4.7
***E. faecalis*** **ATCC 29212**	14.5 ± 1.9	12.9 ± 4.6	10.6 ± 3.9	9.2 ± 2.7	8.3 ± 2.6	5.8 ± 5.2
***K. pneumoniae*** **ATCC13883^T^**	9.9 ± 1.1	9.4 ± 0.9	9.0 ± 0.4	7.1 ± 0.7	6.0 ± 0.0	0
***L. monocytogenes*** **ATCC 15313^T^**	15.4 ± 0.1	12.6 ± 0.8	7.4 ± 0.4	3.5 ± 5.0	3.1 ± 4.4	0
***P. aeruginosa*** **ATCC 27853**	0	0	0	0	0	0
***S. enterica*** **ATCC 14028**	13.3 ± 0.3	11.2 ± 0.7	8.8 ± 0.4	9.3 ± 2.1	7.1 ± 0.1	0
***S. aureus*** **ATCC 25923**	18.7 ± 0.6	16.0 ± 3.1	10.8 ± 0.8	8.8 ± 1.5	7.4 ± 0.1	0
(**B**)						
**Bacterial strain**	**250**	**125**	**62.5**	**31.2**	**16**	**8**
***B. spizizenii*** **ATCC 6633**	10.9 ± 0.9	10.9 ± 0.7	10.2 ± 0.6	8.6 ± 0.9	7.5 ± 0.9	0
***E. coli*** **ATCC 25922**	9.9 ± 2.3	11.1 ± 1.2	9.1 ± 0.3	7.4 ± 0.8	0	0
***E. faecalis*** **ATCC 29212**	10.1 ± 0.7	9.7 ± 2.0	8.6 ± 1.2	2.9 ± 4.0	0	0
***K. pneumoniae*** **ATCC 13883^T^**	9.1 ± 0.6	7.7 ± 0.3	6.8 ± 0.1	0	0	0
***L. monocytogenes*** **ATCC 15313^T^**	12.9 ± 0.2	9.8 ± 0.9	4.3 ± 3.8	0	0	0
***P. aeruginosa*** **ATCC 27853**	6.9 ± 1.0	0	0	0	0	0
***S. enterica*** **ATCC 14028**	11.7 ± 0.5	7.7 ± 0.1	6.4 ± 0.4	0	0	0
***S. aureus*** **ATCC 25923**	15.9 ± 0.3	12.9 ± 0.4	9.8 ± 0.5	7.6 ± 0.4	4.2 ± 3.6	0

**Table 2:** Minimum Inhibitory Concentration (MIC) values for eugenol formulations.

Bacterial Strain	MIC Microparticles	MIC Free Eugenol
	mg/mL	mg/mL
***B. spizizenii*** **ATCC 6633**	1	16
***E. faecalis*** **ATCC 29212**	2	8
***E. coli*** **ATCC 25922**	1	8
***K. pneumoniae*** **ATCC 13883^T^**	1	8
***L. monocytogenes*** **ATCC 15313^T^**	0.5	4
***P. aeruginosa*** **ATCC 27853**	>62.5	>62.5
***S. enterica*** **ATCC 14028**	1	4
***S. aureus*** **ATCC 25923**	1	8

**Table 3:** MIC values of reference antibiotics.

Bacterial Strain	MIC Tetracycline (µg/mL)	MIC Levofloxacin (µg/mL)
***B. spizizenii*** **ATCC 6633**	4	32
***E. coli*** **ATCC 25922**	4	0.06
***E. faecalis*** **ATCC 29212**	32	2
***K. pneumoniae*** **ATCC 13883^T^**	16	1
***L. monocytogenes*** **ATCC 15313^T^**	0.5	16
***P. aeruginosa*** **ATCC 27853**	32	4
***S. enterica*** **ATCC 14028**	16	8
***S. aureus*** **ATCC 25923**	1	0.5

## Data Availability

The original contributions presented in the study are included in the article; further inquiries can be directed to the corresponding author.

## References

[1] O’Neill J. (2016). Tackling Drug-Resistant Infections Globally: Final Report and Recommendations.

[2] World Health Organization (2024). WHO Bacterial Priority Pathogens List, 2024: Bacterial Pathogens of Public Health Importance to Guide Research, Development and Strategies to Prevent and Control Antimicrobial Resistance.

[3] Tacconelli E., Carrara E., Savoldi A., Harbarth S., Mendelson M., Monnet D.L., Pulcini C., Kahlmeter G., Kluytmans J., Carmeli Y. (2018). Discovery, research, and development of new antibiotics: The WHO priority list of antibiotic-resistant bacteria and tuberculosis. Lancet Infect. Dis..

[4] Lewis K. (2020). The Science of Antibiotic Discovery. Cell.

[5] Cowan M.M. (1999). Plant Products as Antimicrobial Agents. Clin. Microbiol. Rev..

[6] Marchese A., Barbieri R., Coppo E., Orhan I.E., Daglia M., Nabavi S.F., Izadi M., Abdollahi M., Nabavi S.M., Ajami M. (2017). Antimicrobial activity of eugenol and essential oils containing eugenol: A mechanistic viewpoint. Crit. Rev. Microbiol..

[7] Jeyakumar G.E., Lawrence R. (2021). Mechanisms of bactericidal action of eugenol against *Escherichia coli*. J. Herb. Med..

[8] Falleh H. (2025). Demystifying the power of essential oils: A review of their antibacterial properties and potential as natural food preservatives. EXCLI J..

[9] Devi K.P., Nisha S.A., Sakthivel R., Pandian S.K. (2010). Eugenol (an essential oil of clove) acts as an antibacterial agent against Salmonella typhi by disrupting the cellular membrane. J. Ethnopharmacol..

[10] Lahiri D., Nag M., Dutta B., Dey S., Mukherjee D., Joshi S.J., Ray R.R. (2021). Antibiofilm and anti-quorum sensing activities of eugenol and linalool from *Ocimum tenuiflorum* against *Pseudomonas aeruginosa* biofilm. J. Appl. Microbiol..

[11] Kerosenewala J., Vaidya P., Ozarkar V., Shirapure Y., More A.P. (2023). Eugenol: Extraction, properties and its applications on incorporation with polymers and resins—A review. Polym. Bull..

[12] Friedman M., Henika P.R., Levin C.E., Mandrell R.E. (2004). Antibacterial Activities of Plant Essential Oils and Their Components against *Escherichia coli* O157:H7 and Salmonella enterica in Apple Juice. J. Agric. Food Chem..

[13] Talón E., Lampi A.M., Vargas M., Chiralt A., Jouppila K., González-Martínez C. (2019). Encapsulation of eugenol by spray-drying using whey protein isolate or lecithin: Release kinetics, antioxidant and antimicrobial properties. Food Chem..

[14] Jafari S.M., Assadpoor E., He Y., Bhandari B. (2008). Encapsulation Efficiency of Food Flavours and Oils during Spray Drying. Dry. Technol..

[15] Gharsallaoui A., Roudaut G., Chambin O., Voilley A., Saurel R. (2007). Applications of spray-drying in microencapsulation of food ingredients: An overview. Food Res. Int..

[16] Dima C., Assadpour E., Dima S., Jafari S.M. (2020). Bioavailability of nutraceuticals: Role of the food matrix, processing conditions, the gastrointestinal tract, and nanodelivery systems. Compr. Rev. Food Sci. Food Saf..

[17] Iesa N.B., Chaipoot S., Phongphisutthinant R., Wiriyacharee P., Lim B.G., Sringarm K., Burgett M., Chuttong B. (2023). Effects of Maltodextrin and Gum Arabic Composition on the Physical and Antioxidant Activities of Dewaxed Stingless Bee Cerumen. Foods.

[18] Zhang H., Dudley E.G., Davidson P.M., Harte F. (2017). Critical concentration of lecithin enhances the antimicrobial activity of eugenol against *Escherichia coli*. Appl. Environ. Microbiol..

[19] Peng S., Zou L., Liu W., Gan L., Liu W., Liang R., Liu C., Niu J., Cao Y., Liu Z. (2015). Storage stability and antibacterial activity of eugenol nanoliposomes prepared by an ethanol injection–dynamic high-pressure microfluidization method. J. Food Prot..

[20] Hyldgaard M., Mygind T., Meyer R.L. (2012). Essential Oils in Food Preservation: Mode of Action, Synergies, and Interactions with Food Matrix Components. Front. Microbiol..

[21] Bouarab-Chibane L., Forquet V., Lantéri P., Clément Y., Léonard-Akkari L., Oulahal N., Degraeve P., Bordes C. (2019). Antibacterial Properties of Polyphenols: Characterization and QSAR (Quantitative Structure–Activity Relationship) Models. Front. Microbiol..

[22] Pesingi P.V., Singh B.R., Pesingi P.K., Bhardwaj M., Singh S.V., Kumawat M., Sinha D.K., Gandham R.K. (2019). MexAB-OprM Efflux Pump of *Pseudomonas aeruginosa* Offers Resistance to Carvacrol: A Herbal Antimicrobial Agent. Front. Microbiol..

[23] Shekunov B.Y., Chattopadhyay P., Tong H.H., Chow A.H. (2007). Particle size analysis in pharmaceutics: Principles, methods and applications. Pharm. Res..

[24] Poole K. (2005). Efflux-mediated antimicrobial resistance. J. Antimicrob. Chemother..

[25] Lister P.D., Wolter D.J., Hanson N.D. (2009). Antibacterial-Resistant *Pseudomonas aeruginosa*: Clinical Impact and Complex Regulation of Chromosomally Encoded Resistance Mechanisms. Clin. Microbiol. Rev..

[26] Lomovskaya O., Warren M.S., Lee A., Galazzo J., Fronko R., Lee M., Blais J., Cho D., Chamberland S., Renau T. (2001). Identification and Characterization of Inhibitors of Multidrug Resistance Efflux Pumps in *Pseudomonas aeruginosa*: Novel Agents for Combination Therapy. Antimicrob. Agents Chemother..

[27] Piddock L.J.V. (2006). Multidrug-resistance efflux pumps: Not just for resistance. Nat. Rev. Microbiol..

[28] Naghavi M., Vollset S.E., Ikuta K.S., Swetschinski L.R., Gray A.P., Wool E.E., GBD 2021 Antimicrobial Resistance Collaborators (2024). Global burden of bacterial antimicrobial resistance 1990–2021: A systematic analysis with forecasts to 2050. Lancet.

[29] Khwaza V., Aderibigbe B.A. (2025). Antibacterial Activity of Selected Essential Oil Components and Their Derivatives: A Review. Antibiotics.

[30] Scalia A.C., Talamo Ruiz J.A., Dalle Vacche S., Bongiovanni R., Lacroix-Desmazes P., Cochis A., Vitale A. (2026). Multifunctional eugenol-enriched PEO-based electrospun and photo-crosslinked scaffolds for wound healing applications. Appl. Mater. Today.

[31] Latorre R., Valerii M.C., Ferrari I., Benati M., Spisni E., Pardo A., Albanese M., Signoretto C., Lippi G., Gaibani P. (2026). Synergistic activity of eugenol, cinnamaldehyde, and carvacrol in combination with different antibacterial agents against multidrug-resistant Gram-negative clinical isolates. Antibiotics.

[32] Clinical and Laboratory Standards Institute (2018). Methods for Dilution Antimicrobial Susceptibility Tests for Bacteria That Grow Aerobically.

[33] Balouiri M., Sadiki M., Ibnsouda S.K. (2016). Methods for in vitro evaluating antimicrobial activity: A review. J. Pharm. Anal..

[34] Hartman K., Świerczyńska M., Wieczorek A., Baszuk P., Wojciechowska-Koszko I., Garbacz K., Sienkiewicz M., Kwiatkowski P. (2025). Preliminary Evaluation of the Synergistic Antibacterial Effects of Selected Commercial Essential Oil Compounds Against Methicillin-Resistant Staphylococcus aureus ATCC 43300. Antibiotics.

